# Domestic Correspondence

**Published:** 1881-08

**Authors:** 


### Domestic Correspondence.

ARTICLE XIII.

FALLS CITY, NEB., June 6, 1881.

EDITORS MEDICAL JOURNAL AND EXAMINER: My attention was first called to the term, gangliasthenia, by an article in the *Michigan Medical News*, under date of April 11, 1881, written by Dr. E. Halsey Wood, of Hersey, Michigan; and according to this gentleman, "gangliasthenia means loss or diminution of ganglionic nerve power." "The term does not denote the condition of the ganglia, but expresses the fact that they do not generate a normal amount of nerve force."

Previous to the appearance of Dr. Wood's article I had seen and treated several cases of a peculiar nervous trouble, which seemed to be affections of the sympathetic system of nerves, as indicated by the symptoms.

In meeting with these cases, I was puzzled to know what to call them, and did not call them by any name, except that vague term "nervous trouble," which satisfies the people, and we can go on and treat. But this did not satisfy me, and ought not to satisfy any physician.

Upon looking for something on the subject, in our text books, I could find nothing in the books to which I had access; so I treated them according to common sense, and fulfilled the indications which the symptoms pointed out. Upon seeing Dr. Wood's article, it gave me a name which I thought was a good one for the trouble or disease I had met with, and which I think, will be plainly seen by the history of the following case:

On February 9, 1881, was called out five miles from town, to see a Mr. H. Upon asking the man who came for me (who was his brother), what seemed to be the trouble, he said his brother was suffering with chills at the present, but had been sick for several months, and did not know what was the matter. He had been treated during that time, first by a couple of physicians from a neighboring town, and last by an eclectic physician from this place; but the patient did not improve. Mr. H.'s trouble had been called kidney disease, liver trouble, chills and ague, heart disease, dyspepsia, and his friends thought he was dying of consumption. Upon reaching the house, I found the patient to be a young man about twenty-five years of age; an American; had been married about seven months; a farmer; had been very healthy until about three months previous. The first symptom he noticed was his becoming very tired upon slight exercise; his back ached, he was languid; he did not rest well at night; was troubled with constipation, and with frequent frontal headache; his food was not digested well, and sometimes gave pain; his appetite was changeable; he became nervous and weak and began to lose flesh; was troubled with palpitation of the heart, slight cough, frequent flushes or rush of blood to different parts of the body, with a sensation of warmth or heat; frequent chilly sensations; localized sweating, such as one limb, the body, the hands or the head and face; insomnia; frightful dreams; blurred or disordered vision, congested conjunctivæ, convulsive closure of the extremities, and frequent epistaxis. These symptoms, slight at first, continued and gradually increased in severity. When I saw him, February 9, he was confined to his bed, suffering with se ere palpitation of the heart, one or two severe chills a day, without much fever after them, sometimes he would have quite high fever without any marked chill; he had pain over the heart and liver; headache; sleep very much disturbed, and so nervous that if a person should touch the bed-covers it would startle him, even if he was asleep; his back was very weak; his bowels very irregular, sometimes constipated and sometimes troubled with diarrhœa; at this time he had no localized sweating, but would sweat all over very profusely, from three to seven hours at a time; would wet the bed-clothes and sometimes through the mattress.

I examined his heart and lungs and found them healthy, except with slight bronchitis, his cough was sometimes dry and sometimes with free expectoration. I examined his urine, and found his kidneys in a healthy state, but his urine was at times free and abundant, while at other times scanty.

I will now leave the readers of this article to draw their own conclusions as to the relation of the sympathetic system of nerves and the foregoing symptoms, and the propriety of calling this trouble "gangliasthenia."

As regards treatment. I first had him give up the use of strong tea, of which he drank three or four cups a day; also the use of tobacco, of which he used a great deal; had him take a tablespoonful each of Scott's emulsion of pure cod-liver oil and Trommer's extract of malt, three times a day, and bromide of ammonium and potassium, each seven grains, tr. ferri chlorid. eight drops, and tr. digitalis, six drops every three hours. Sulphate of atropia, 1-80 of a grain, to be taken when the profuse sweating began. When his bowels were costive or constipated to take a teaspoonful of the following, night and morning:

℞. Fl. ext. cascaræ sagradæ.

Tr. aloes et myrrh.

Syr. tolu, aa ℥j.

M.

Saw the patient again on the 15th, and found him much better; he felt stronger, was not so nervous, the chills were not so frequent nor so severe; his heart did not palpitate as much; his head ached very little; did not sweat so much; rested a little better at night, and did not dream so much; his nose did not bleed as often; appetite still poor; vision blurred; congested conjunctiva; still convulsive closure of the eye-lids; coldness of extremities and flushes of heat less frequent; cough no better. Continued the treatment with the addition of ten grains of chloral hydrate at bed-time.

March 2, was called to see Mr. H.; the family thought he was dying. I found him very much excited, respirations forty-eight in a minute, with considerable pain in the chest, skin dry and extremities cold. Gave him some brandy, with about twenty drops of fl. ext. of jaborandi, and had his feet bathed in hot mustard water. In about fifteen or twenty minutes he began to perspire quite freely; his respirations soon came down to twenty-four per minute. For two or three days after, cough was worse and the sputa were rust colored, and the first day streaked with blood, indicating more or less congestion of the lungs. Continued the same treatment with an increase of ammonium bromide to ten grains. I did not see him again until March 24, at which time his cough had left; he could sit up all day; his appetite was better but digestion painful; his bowels were regular; profuse sweating had ceased; still had chilliness, but no marked chills; rested well at night, had no bad dreams nor headache; had no convulsive closure of the eyelids, vision was good; the hot flushes were very slight; had no palpitation of the heart; was still nervous and weak. Continued the oil and malt, gave citrate of quinine and iron ten grains, bromide of ammonium ten grains, and potassium bromide five grains three times a day, ten grains of lactopeptine after each meal, and cold salt water baths every other day, followed by rubbing with a coarse towel. This treatment was continued until April 5, when he was feeling about well, was able to be out of doors, but was still feeling weak. I withdrew all medicine, except the oil and citrate of quinine and iron, which he took twice a day, until April 15, when all medication was stopped, and to-day he feels better than he did before he became sick, but not as strong.

A. B. NEWKIRK, JR., M.D.

---

### ARTICLE XIV.

EDITORS CHICAGO MEDICAL JOURNAL AND EXAMINER:—On April 17 I delivered Mrs. Rebecca Denning of an eleven-pound boy—full time. Mrs. D. is a native of Clay county, Indiana, where she was born the 13th day of January, 1827. She was fifty-four years three months and four days old at the time of her last delivery. She is the mother of thirteen children. She is a large woman, weighing about 200 pounds, of a sanguine, lymphatic temperament. Mr. John Denning, her husband, is sixty-four years past, hale and active, physically and mentally.

Yours cordially,

STREATOR, ILL., May 14, 1881. JNO. J. TAYLOR.

### ARTICLE XV.

EDITORS CHICAGO MEDICAL JOURNAL AND EXAMINER:—I was delighted to read the excellent paper of Dr. Clevenger in the May number of your splendid journal, on the labors of my old friend and classmate, Dr. Schmidt. Allow me to mention another piece of ingenious mechanism of his I saw utilized for a most excellent purpose, whilst I was taking my degree course in the Medical Department of University of Pennsylvania, whilst the Doctor was prosector for Dr. Leidy, which to me is entirely unique.

He took an articulated skeleton, and I am not sure but that he made that also, and on one side he sawed in two the bones of the extremities and fastened them together with a screw, and then made muscles of india rubber and attached them naturally, so by un-screwing it represented a fracture, and demonstrated the muscles causing the deformity; on the other side of the same skeleton were like muscles to show the deformity of luxations; this was for Dr. H. H. Smith, Professor of Surgery, and on me at least, they made a lasting impression, as I have never forgotten them.

The last time I had the pleasure of seeing the Doctor was in Selma, just after the surrender. I too had been Assistant Surgeon in the Confederate army, and like the Doctor, reduced from affluence to poverty. I was living in Alabama, my native State, not long afterward removing to this State, and I had lost sight of the Doctor, until reading Dr. Clevenger's paper, before referred to. All honor and a long life to the Doctor, for his devotion to our noble profession. R. FOWLER, M.D.

AURORA, TEXAS, May 21, 1881.

---

PROFESSOR I. N. DANFORTH, of Chicago, has recently received from his Alma Mater, Dartmouth, the honorary degree of Master in Arts, a distinction which he has long deserved and which will in the future be succeeded by higher honors, if they who know him are not mistaken.

